# Alzheimer’s and Parkinson’s disease drugs side effects

**DOI:** 10.1192/j.eurpsy.2024.1312

**Published:** 2024-08-27

**Authors:** J. Batistela, F. J. Ropero Pelaez, G. Hida, S. Taniguchi

**Affiliations:** ^1^Albert Einstein Hospital, São Paulo; ^2^Mathematics, Computation and Cognition, University Federal of ABC, Sao Paulo; ^3^Big Data Analytics - Artificial Intelligence, Albert Einstein Hospital; ^4^São Paulo University, São Paulo, Brazil

## Abstract

**Introduction:**

Alzheimer’s and Parkinson’s disease are neurodegenerative disorders with life limiting conditions. The symptomatic pharmacological therapeutic strategies unfortunately are also related to undesirable side effects. Acetylcholinesterase inhibitors administered to Alzheimer’s disease patients increase cholinergic transmission in cortex and hippocampus.

Antiparkinsonian drugs increase dopaminergic system activity, to compensate for dopaminergic neurons’ degeneration in *corpus striatum, t*herefore supplying the imbalance of these neurotransmitters in these degenerative areas.But undesirable the increase of these neurotransmitters in other cerebral and peripheral areas brings us important side effects

**Objectives:**

To study Alzheimer’s cholinergic drugs and Parkinson’s dopaminergic drugs’ side effects

**Methods:**

This retrospective study included 107 geriatric patients enrolled in a private long-term care institution. 79 patients with Alzheimer’s disease had mean age of 88.11 ± 5,78 years old, mean weight of 61.62 ± 13.10 kg. 28 patients with Parkinson’s disease had mean age of 84.93 ± 5.71 years old, weight mean 66.36 ± 2.83 kg.

**Results:**

Alzheimer’s disease patients 41.77% (33) received. Acetylcholinesterase inhibitors (Donepezil, galantamine and rivastigmine) Psychomotor agitation and aggressive behavior 63.666% and nausea (15%) were observed in the patients treated with these drugs. The association of L-DOPA and DOPA decarboxylase inhibitors (benserazide) were administered to 53%(15) of the Parkinson’s disease patients in doses between 2.0-19.0 mg/kg/day. L-DOPA associated to catechol-O-methyltransferase inhibitor (entacapone) 3 mg/kg/day were given to 7.14% (2) patients. Bromocriptine 0.04 mg/kg/day was given to 3.57% (1) patients. Mental confusion and hallucination side effects were observed in 53.33% (8) patients treated with L-DOPA associated with the DOPA decarboxylase inhibitor (benserazide).

**Conclusions:**

The increase of cholinergic activity due to the acetylcholinesterase inhibitors in the Nigro- striatal pathway could be related to psychomotor agitation in Alzheimer’s disease patients in a similar way to akathisia induced by neuroleptics. The increase of dopamine levels due to the administration of L-DOPA, in *corpus striatum* improved Parkinson’s disease symptoms although the increase of dopaminergic activity at mesocortical pathways may be related to confusion and hallucination observed in these patients. Adjustments in dosage of these drugs could provide improvement in these patients’ daily life conditions.

**Disclosure of Interest:**

None Declared

